# NO in Viral Infections: Role and Development of Antiviral Therapies

**DOI:** 10.3390/molecules27072337

**Published:** 2022-04-05

**Authors:** Federica Sodano, Elena Gazzano, Roberta Fruttero, Loretta Lazzarato

**Affiliations:** 1 Department of Drug Science and Technology, University of Torino, 10125 Torino, Italy; roberta.fruttero@unito.it (R.F.); loretta.lazzarato@unito.it (L.L.); 2 Department of Pharmacy, “Federico II” University of Naples, 80131 Naples, Italy; 3 Department of Life Sciences and Systems Biology, University of Torino, 10123 Torino, Italy

**Keywords:** nitric oxide, viral infections, NO-donors, inhalation therapy, COVID-19

## Abstract

Nitric oxide is a ubiquitous signaling radical that influences critical body functions. Its importance in the cardiovascular system and the innate immune response to bacterial and viral infections has been extensively investigated. The overproduction of NO is an early component of viral infections, including those affecting the respiratory tract. The production of high levels of NO is due to the overexpression of NO biosynthesis by inducible NO synthase (iNOS), which is involved in viral clearance. The development of NO-based antiviral therapies, particularly gaseous NO inhalation and NO-donors, has proven to be an excellent antiviral therapeutic strategy. The aim of this review is to systematically examine the multiple research studies that have been carried out to elucidate the role of NO in viral infections and to comprehensively describe the NO-based antiviral strategies that have been developed thus far. Particular attention has been paid to the potential mechanisms of NO and its clinical use in the prevention and therapy of COVID-19.

## 1. Introduction

The enormous cost of viral infections has been made abundantly evident over the last two years, in which we have observed the worldwide spread of severe acute respiratory syndrome coronavirus-2 (SARS-CoV-2), which affects every aspect of human life and all economic sectors [1]. After the outbreak of SARS-CoV-1, in 2002, and of Middle East respiratory syndrome coronavirus (MERS-CoV), in 2012, SARS-CoV-2 has infected over 326 million people since 2019, causing the death of nearly 6 million (17 February 2022, source Johns Hopkins Center for Systems Science and Engineering, https://coronavirus.jhu.edu/map.html). Unfortunately, the availability of effective antiviral drugs is limited. Although a great deal of effort has been devoted to the development of new therapeutic approaches since the first approval of an antiviral drug in 1963, only 90 drugs were formally approved up to 2016 [2]. Among the difficulties in antiviral drug research, we find the great variety of viral species and their fast mutation rate, which leads to the development of resistance, greatly reducing the efficacy of existing therapies [3]. Resistance to antiviral drugs is a major public health issue and has led to increased mortality, poor quality of life, and enormous social and economic costs [4]. Furthermore, antiviral drugs often also affect host biological processes, further limiting their clinical efficacy. For all of these reasons, the development of new strategies to tackle viral infections is an urgent need and nitric oxide (NO) is an effective approach to this end. NO plays an important role in both cardiovascular and immune systems [5,6], and its antibacterial activity has been well described [7]. Its use against viruses has more recently been proposed and NO-based therapeutic strategies have been investigated [8]. The aim of this review is to describe the proposed antiviral mechanisms of NO and to present NO-based therapies that can be effective against viral infections. Finally, the factors limiting the clinical translation of this approach will be commented upon.

## 2. Biology of NO

NO is a radical species that is involved in a variety of physiological and pathological processes. It is ubiquitous in mammalian tissues, where it is produced from the conversion of L-arginine into L-citrulline in a reaction catalyzed by three isoforms of the enzyme NO-synthase (NOS). The three isoforms differ in terms of the regulation, amplitude and duration of NO production, and their tissue localization [6]. Neuronal (nNOS) and endothelial (eNOS) NOS are constitutive enzymes found in neuronal and skeletal muscle tissues and vascular endothelial cells, respectively; their activation is calcium-dependent and produces NO on a timescale from seconds to minutes, giving rise to low NO concentrations (pM-nM) [9,10]. The main physiological roles of NO within the vasculature are the inhibition of platelet activation and therefore the prevention of thrombosis and vasodilation [8]. In the nervous system, NO exerts its role by acting as a neurotransmitter and via its vasodilatory properties [11]. Inducible NOS (iNOS) can work in a calcium-independent manner and its activity is associated with immune functions. It is found in macrophages, monocytes and muscle cells, where it produces NO on a timescale from hours to days, leading to high concentrations of the molecule (μM), making NO an important weapon in the immune response against bacteria, viruses and cancer cells [9]. In the immune system, NO is not only produced by activated macrophages but also by dendritic cells, natural killer cells, mast cells and other immune-system cells [6]. The constitutive expression of iNOS has recently been described as occurring in lung epithelial cells, where the enzyme seems to be the principal source of exhaled NO, with activation being calcium-dependent in this case [12]. An alternative synthetic pathway for NO formation, independently of classical NOS enzymes, has been described as passing through the reduction of nitrites thanks to the nitrate reductase activity of xanthine oxidase. This route seems to be particularly important when endothelial functions are reduced due to aging or cardiovascular diseases [13].

NO has direct effects in that it can block the functions of target molecules by reacting with the metal centers (e.g., iron-sulfur centers of proteins and enzymes). It also has indirect effects that are mediated by its reaction with oxygen and the superoxide anion to give reactive nitrogen species (RNS), which then oxidize, nitrate and nitrosate target molecules [14].

The balance between the physiological and pathological effects of NO depends on its concentration, meaning that any proposal to use NO for therapeutic purposes should take its concentration and localization into careful consideration.

## 3. Role of NO in Immune Defense against Viruses

While endogenous NO is one of the major mediators in the body’s defense against microorganisms in the airway, its antiviral role has been less extensively studied. The non-specific action that NO exerts against viruses was first described in the early 1990s, and, since these pioneering studies, further evidence has demonstrated that NO exerts an action against all types of viruses, including the coronavirus, with broad activity [14,15]. Research in the coronavirus field intensified after the emergence of a new human pulmonary disease in 2002, severe acute respiratory syndrome (SARS), which was caused by a novel coronavirus, SARS-CoV-1. In 2004, the NO donor *S*-nitroso-*N*-acetylpenicillamine (SNAP) was demonstrated to have in vitro activity against SARS-CoV-1 [16]. Subsequently, Akerstrom et al. demonstrated the mechanisms by which NO can inhibit the replication cycle of SARS-CoV-1 [17,18]. Studies into NO activity against coronavirus have received even further impetus since the recent outbreak of SARS-CoV-2.

**Figure 1:**
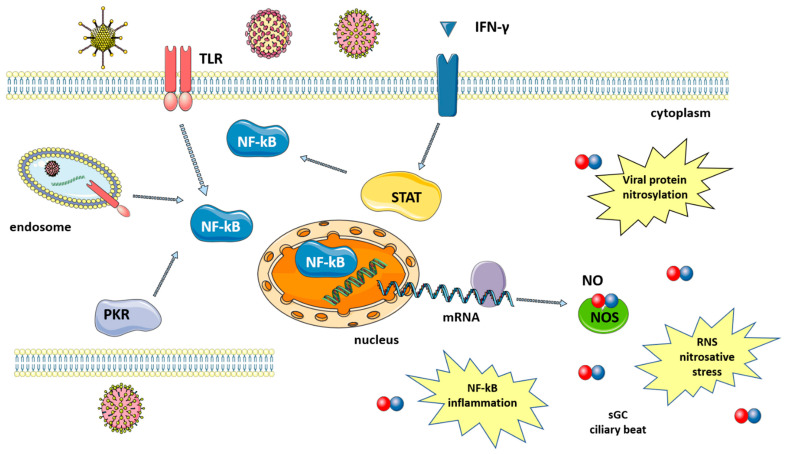
Synthesis of nitric oxide after viral infection. Viruses induce signaling pathways that converge in the activation of NF-kB and iNOS expression. Levels of NO increase, leading to viral protein nitrosylation, nitrosative stress, induction of an inflammatory response, increased ciliary beat, etc. Signaling pathways are described in the text. The figure was created by modifying images obtained from Smart Servier Medical Art (17 February 2022, smart.servier.com) licensed under a Creative Commons Attribution 3.0 Unported License.

Many studies have shown that a number of diseases caused by viral infection are characterized by an increase in NO levels. Indeed, following infection, the large amounts of NO that are necessary to fight viruses are produced thanks to the upregulation of iNOS via the activation of several pathways that are mediated by: (i) toll-like receptors (TLRs); (ii) signal transducer and activator of transcription 1 (STAT-1); and, (iii) protein kinase-R (PKR) [8], as summarized in Figure 1. TLRs are expressed in immune (macrophages, lymphocytes) and non-immune (epithelial) cells where they detect pathogen-associated molecular patterns (PAMPs), which, in the case of viruses, can be the viral nucleic acid or components of viral envelopes. The recognition of PAMPs by TLRs results in the activation of signaling pathways that converge in the activation of transcription factors, nuclear factor kappa-light-chain enhancer of activated B cells (NF-κB) and activator protein 1 (AP-1), which enters the nucleus and upregulate iNOS transcription [19]. A second pathway is mediated by interferon-gamma (IFN-γ), which is produced by immune cells around 24–26 h after infection. IFN-γ induces the phosphorylation and activation of Janus kinase 1 (Jak1), which, in turn, phosphorylates and thus activates STAT-1, leading again to the upregulation of iNOS [20]. Finally, double-stranded RNA-dependent protein kinase (PKR), activated by viral infections, plays a major role in the activation of iNOS through the NF-κB pathway [21].

iNOS activation results in the increased production of NO, which is effective against both DNA and RNA viruses, including SARS-CoV-2 [22]. NO action against viruses proceeds via several different mechanisms, both direct and indirect, which will be briefly discussed below (Figure 1).

(1) The inactivation of viral proteins via nitrosylation reduces or inhibits enzyme activity. Target proteins include proteases, reverse transcriptases, ribonucleotide reductases, transcription factors and tyrosine-containing enzymes [19]. The mechanism is the *S*-nitrosylation of the cysteine residues that are directly involved in catalysis or present near the active site [15]. For SARS-CoV in particular, this virus encodes two cysteine proteases that are known to be particularly susceptible to nitrosylation [18]. In fact, a decrease in SARS-CoV-2 protease activity has been reported after treatment with a NO-donor and was explained as acting via the *S*-nitrosylation of the cysteine in the enzyme active site [23]. Host proteins can also be nitrosylated, and the reaction between NO and either cysteine or thiols is not always detrimental, with it being able to help innate immune response in some cases [24]. Another mechanism for NO-mediated SARS-CoV protein inactivation occurs via the reduced palmitoylation of the spike (S) protein, which affects the interaction between the S protein and the ACE2 receptor [18].

(2) Deamination of viral DNA following nitrosative stress. NO induces DNA damage both to viral and host genetic material, although host DNA can be repaired by the DNA repair machinery [8].

(3) NO increases the clearance of pathogens, including DNA and RNA viruses, by enhancing ciliary beat frequency [8,25]. NO activates soluble guanylyl cyclase (sGC) in airway epithelial cells to produce cyclic guanosine monophosphate (cGMP), which, in turn, activates protein kinase G (PKG). PKG phosphorylates effector proteins leading to an increase in ciliary beat frequency [26].

The high levels of NO produced following iNOS induction also play a role in the inflammatory response. Acute inflammation is a defense mechanism against pathogens and has the aim of destroying or inactivating the invading microorganisms. If microbes are killed and cellular debris removed, inflammation is resolved and the normal tissue is restored. However, when excessive and non-regulated inflammation persists, it leads to tissue damage. During sustained inflammation, NO is produced in high quantities and regulates the activity of a variety of inflammatory mediators, including NF-kB [22]. Other effects of NO include increased blood flow and vascular permeability, further contributing to inflammation [27]. In some viral pathologies, excessive levels of NO are associated with more severe disease course, as has been described in influenza [28], and HIV [29].

## 4. Role of NO in Vascular Endothelium

The endothelial cells that line vessels control several vascular functions, including blood flow, blood fluidity and vascular permeability. For an in-depth description, see the review by Pober and Sessa [27].

The vascular endothelium acts in response to changes in the microenvironment (i.e., oxygen concentration) and induces the release of specific mediators that act in the control of the tone of smooth muscle cells [27]. These molecules are either vasodilators (such as NO and prostacyclin) or vasoconstrictors (such as endothelin), which exert their action on vascular smooth muscle cells. NO, which promotes vasodilation, is one of the most important of these mediators in the cardiovascular system. The NO produced in low levels by endothelial cells is fundamental for normal vascular tone. The mechanism at the base of the vasodilatory effect is the activation of sGC and the production of cGMP. The reduction of NO, which leads to impaired sGC–cGMP signaling, is associated with vascular dysfunctions, including hypertension and other cardiovascular diseases [30].

Under physiological conditions, the maintenance of blood fluidity, via the inhibition of coagulation is another role of the vascular endothelium [27]. NO also plays a crucial role here by acting as an anticoagulant with a mechanism that again involves the activation of the NO–sGC–cGMP signaling pathway [14]. The synthesis of NO is activated by many stimuli, including shear stress, variations in Ca^2+^ concentration and mediators such as acetylcholine and bradykinin [31]. NO works in synergy with prostacyclin and inhibits platelet adhesion and activation [27].

Impaired endothelial function, with reduced NO availability, is found in diseases such as diabetes mellitus, obesity and hypertension [32]. The reduction in NO production is probably involved in the pathogenesis of thrombosis [33].

### Vascular Endothelium Dysfunctions Associated with Viral Infections

The maintenance of endothelium functionality is essential as it allows for controlled exchanges between blood and the surrounding tissue and corrects vascular tone, while also avoiding the formation of a pro-thrombotic environment. Alterations in the vascular endothelium are therefore associated with pathological conditions, such as those related to viral infections. HIV infection is associated with oxidative stress, which reduces NO availability, leading to endothelium dysfunctions [34]. Similarly, emerging viruses that pose a relevant threat to human and animal health, including SARS-CoV-2, may also affect endothelial cell functions, as recently reviewed in [35]. Coronavirus-like SARS-CoV-1, MERS-CoV and SARS-CoV-2, which have been shown to infect endothelial cells, lead to a decrease in endothelial NO [14]. Indeed, SARS-CoV-2 infection seems to disrupt the signaling for endothelial NO production [36] and significantly decreased endothelial NO levels were measured in patients with COVID-19 [37]. Angiotensin-converting enzyme 2 (ACE2), which is the virus gateway into cells, converts angiotensin II to angiotensin 1-7, activating a pathway that increases NO production, among other effects. Virus infection inactivates this route, and, as a consequence, lowers the availability of NO [36], which has been hypothesized as one of the reasons for COVID-19 deaths [38]. During pathological processes that involve endothelial cell dysfunctions, NO levels decrease, leading to vasoconstriction, impaired blood flow and hypercoagulation. Abnormal coagulation has been reported and associated with poor prognosis in patients infected with SARS-CoV-1 and MERS-CoV [14]. Infection from SARS-CoV-2 is also associated with altered coagulation, and a higher risk for thromboembolism has been observed [39]. This aspect can be exploited in therapy with NO, thanks to its effects on vascular endothelium.

## 5. NO and the Respiratory System

NO is produced in the human airway by the three isoforms of NOS [20]. A reduction in lung NO levels is found in diseases such as cystic fibrosis and acute respiratory distress syndrome [20]. In the lungs, NO that is produced by eNOS in endothelial cells improves oxygenation; it acts on smooth muscle cells, inducing vessel dilation and, therefore, an increase in local blood flow [40]. The main mechanism of vasodilation is the same as was described in the previous paragraph, with the involvement of sGC and cGMP. A reduced sGC expression, which is related to decreased vasodilation in response to NO, is often found in adult lungs with respect to children [40]. In addition, NO acts also through cGMP-independent mechanisms: it has been reported that direct activation of Ca^2+^-dependent K^+^ channels [41] and the involvement in the formation of nitrosothiols, which exhibit bronchodilator activity [42].

Another factor in the respiratory system is the lung surfactant, a complex of proteins and phospholipids with the function of reducing surface tension at the air/liquid interface: evidence suggests a role for NO in surfactant secretion and the preservation of its function [43]. At low doses, NO can scavenge radicals, inhibiting lipid peroxidation and protecting lung surfactant structure [43]. At higher concentrations, NO-driven *S*-nitrosylation of surfactant proteins can induce a conformational change, leading to inflammatory response [24].

iNOS, which is constitutively expressed in the airway epithelium, also contributes to the production of NO, which, among other functions, helps the regulation of ciliary beat, a defense mechanism of airway epithelium [12]. The effects of NO on ciliary motility have been demonstrated with the use of NOS inhibitors [43] and by the fact that NOS isoforms are expressed in the ciliary epithelium [44].

The use of NO in the treatment of pulmonary diseases is derived from this evidence [45].

## 6. NO-Based Antiviral Strategies

Although the role of NO in inhibiting viral replication in host defense has been demonstrated, NO-based antiviral strategies have not yet been extensively investigated. To date, no systematic approach has been adopted for the study of NO-based antiviral therapeutics, apart from those active in the cardiovascular system [46,47]. Despite inconsistencies, many researchers have attempted, in reviews, to comprehensively and systematically describe the therapeutic applications of NO following viral infections. Reiss et al. reported the in vitro and in vivo studies that were carried out up to 1998 on NO following viral infection [48], whereas Garren et al. provided a very useful review on the most recent advances in NO-based antiviral therapies [8].

NO-based antiviral strategies can be mainly classified into two groups: gaseous NO (gNO) inhalation-based therapies and NO-donors. As well as chronic inflammatory diseases, viral infections of the upper respiratory tract are related to the presence of higher levels of NO in the air exhaled by human subjects. This is due to an increase in NO production as part of the host response to infection [49], with the increase in the endogenous synthesis of NO being caused by iNOS overexpression that should be involved in viral clearance. Most studies have demonstrated the efficacy of gNO therapies as well as NO-donors in inhibiting RNA replication in viral strains. A schematic summary of clinical trials as well as in-vivo, in-vitro and ex-vivo studies on the use of gNO and NO-donors as active antiviral agents are reported in Table 1.

### 6.1. gNO Inhalation

The greatest efficacy found in gNO therapy to date has been demonstrated in the treatment of oxygen desaturation in infants with hypoxic respiratory failure. The treatment has been approved as a drug by the FDA under the trade name INOMAX^TM^; the recommended dosage was 20 ppm and the length of treatment was up to two weeks.

In the antiviral field, gNO inhalation-based therapies have resulted in quite discordant data, with one of the reasons for this being the gNO dosage sensitivity threshold. This limitation has been highlighted in gNO treatments following acute respiratory distress syndrome (ARDS), which is a particular pathological condition with a number of different etiologies such as SARS-related coronavirus infection (SARS-CoV), pulmonary ischemia-reperfusion (I/R) injury and bacterial/viral pneumonia. The role of NO in animal and human subjects affected by ARDS has been described in a work by Chen et al. [65].

In a Beijing rescue study of fourteen patients with ARDS, which manifested as a result of SARS-CoV infection, gNO-based treatments performed for at least three days at a dosage of 30 ppm led to a marked improvement in arterial oxygenation in patients and a concomitant decrease in pneumonia infiltrates [50]. On the other hand, in some in vivo models of acute lung injury in mice [66,67], gNO increased I/R-induced lung damage and thus became a concomitant cause of endotoxin-induced ARDS. In clinical studies, gNO inhalation was seen to provide no benefit in ARDS-refractory hypoxemia [68,69] and severe influenza. Even intermittent gNO treatment at a dosage of 160 ppm was unable to reduce viral load in the lungs of in vivo mouse models [54].

Although the dosage of gNO in antiviral treatments must be significantly higher than in other applications, e.g., antimicrobial treatment [49,66,70,71], gNO therapy causes weak deleterious effects because of the short half-life of NO [72,73].

Other discrepancies in gNO inhalation-based antiviral therapies have been identified in dosage and the dependence of the therapeutic efficacy on the mode of gNO replenishment. In the antimicrobial field, Hall et al. observed a huge gap in the dosage between gNO and a soluble NO-releasing chitosan oligosaccharide [74]. In other studies, the fluctuation in antimicrobial response was due not only to the short half-life of gNO but also to the difficulty in the phase transfer of gNO to the physiological medium [75]. These limitations certainly did not apply to antimicrobial NO-donors, which instead exhibit greater stability, easier application and controlled metabolic degradation. The weaknesses of gNO inhalation-based therapy can also be found in the antiviral field in addition to in antimicrobial applications, meaning that interest in the use of NO-donors as antiviral agents must be enhanced.

### 6.2. NO-Donors

**Figure 2:**
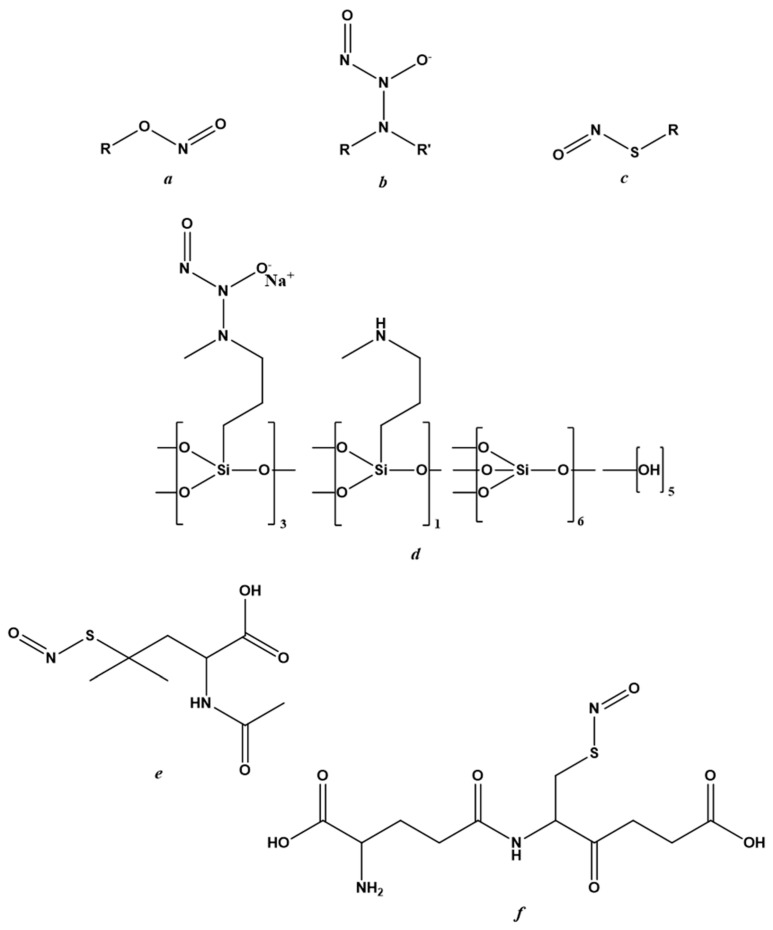
Chemical structure of NO-donors. (**a**) nitrites; (**b**) *N*-diazeniumdiolates; (**c**) *S*-nitrosothiols; (**d**) berdazimer sodium; (**e**) *S*-nitroso-*N*-acetylpenicillamine; (**f**) *S*-nitrosoglutathione.

There are three classes of NO-donors with antiviral effects; nitrites, *N*-diazeniumdiolates and *S*-nitrosothiols (RSNOs). Their general chemical structures are reported in Figure 2a–c, respectively. Despite causing side effects including skin discoloration and irritation [52], the first clinical trial was performed on acidified nitrites in topical ointments against molluscum contagiosum infection. Specifically, these were ointments containing 5% sodium nitrite co-applied with 5% salicylic acid.

NVN1000 (berdazimer sodium, CAS 1846565-00-1) (Figure 2d), which is a compound belonging to the *N*-diazeniumdiolates class and that has antiviral effects in Human Papillomavirus (HPV) raft cultures [61], also displayed efficacy against molluscum contagiosum infection. This NO-donor has been marketed as SB206 by NOVAN Inc., Morrisville, NC, USA, both for application against molluscum contagiosum and in the presence of HPV infection. Three clinical trials are underway: the first (NI-MC201) in phase II for HPV application; the other two (NI-MC301 and NI-MC302) in phase III against molluscum contagiosum [51,53,76].

SNAP (Figure 2e), a derivative of RSNO, has displayed antiviral potential in many in vitro models of viral infection [17,48,60,77,78,79], including a model of SARS-CoV-2 infection [23]. In addition to its high stability under physiological conditions, SNAP showed no cytotoxic in vivo effects in either the bloodstream or the urinary tract.

Several NO-donors, including SNAP and *S*-nitrosoglutathione (GSNO), have been incorporated into polymeric platforms [80,81,82]. The use of these macromolecular scaffolds was necessary to improve the physicochemical profiles of NO-donors and, thus, NO-delivery and targeting [81,83,84]. The architecture of the nanosystem, its biodegradation mechanism and the stimulus causing NO release were designed around the properties of the NO-donor to be incorporated and the organ or tissue to be targeted [83,84,85,86,87,88]. The rational development of NO-releasing nanoformulations in the antiviral field must take into account the stage of viral infection at which it is intended to act.

### 6.3. Endogenous NO-Regulating Drugs

**Figure 3:**
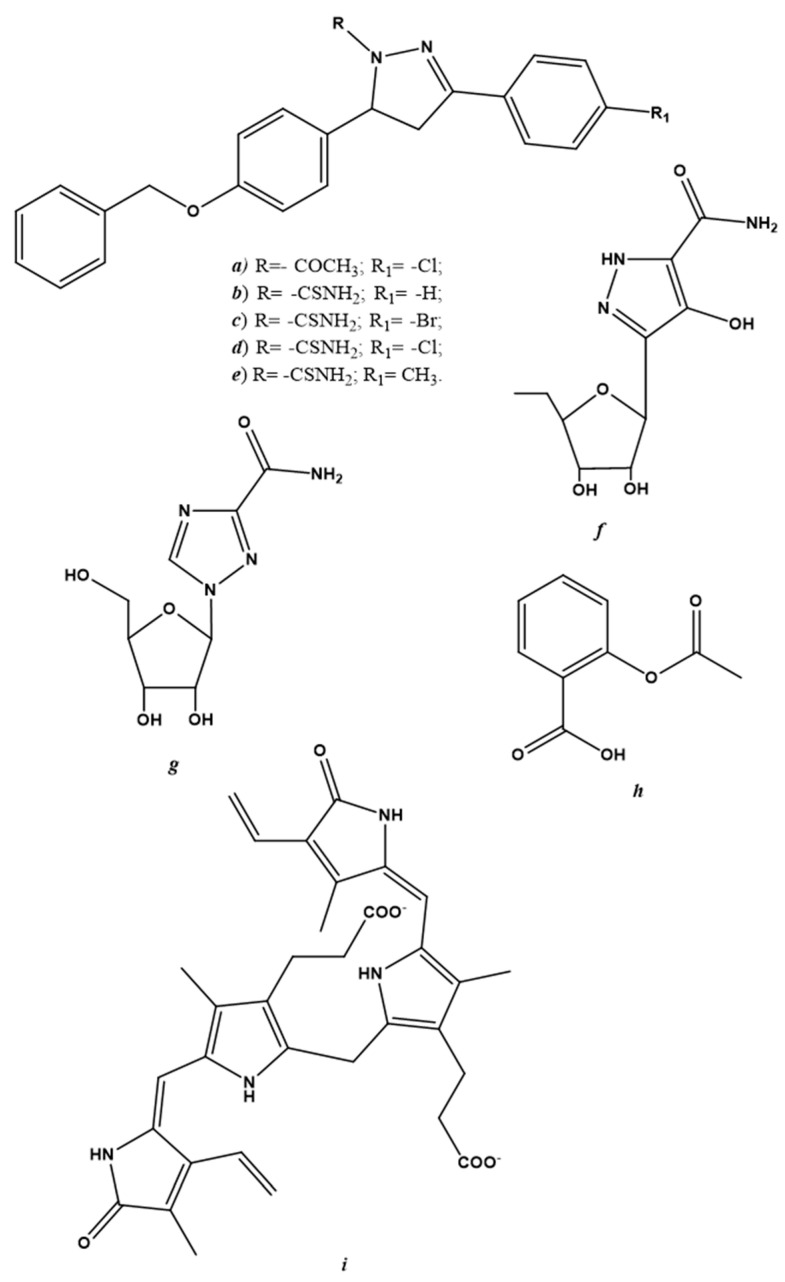
Chemical structure of endogenous NO-regulating drugs. (**a**–**f**) pyrazole derivatives; (**g**) ribavirin; (**h**) acetylsalicylic acid; (**i**) bilirubin.

Endogenous NO-regulating drugs may be considered another potential class of antiviral drugs. These derivatives are described separately from gNO inhalation and NO-donors because of several discordances in the data collected in the literature. Reducing endogenous NO production is undoubtedly a therapeutic antiviral strategy and can be achieved by modulating iNOS activity. Pyrazole derivatives are examples of selective iNOS inhibitors that are active against the vaccinia virus [89,90,91]; chemical structures are shown in Figure 3a–f.

Already-known drugs, such as ribavirin (Figure 3g) have been used to treat respiratory syncytial virus (RSV) infection, hepatitis C and some viral hemorrhagic fevers (e.g., Lassa fever, Crimean-Congo hemorrhagic fever and Hantavirus infection) have shown a two-fold decrease in NO levels in cultured macrophages [92]. Acetylsalicylic acid (Figure 3h) is another widely-used drug that is capable of decreasing hepatitis C virus expression [93]. In addition to its anti-inflammatory power, acetylsalicylic acid has exhibited antiviral activity via the modulation of iNOS [94].

Not always regulating iNOS activity in order to decrease endogenous NO production has led to the arrest of viral replication. In specific cases, instead of regressing, viral infection advanced if iNOS activity was downregulated. An example of this can be found in a study carried out by Santangelo et al. [95]; the virucidal activity of bilirubin (Figure 3i), following herpes simplex virus-1 and enterovirus infection in the Hep-2 and Vero cell lines, was due to its ability to enhance iNOS activity and, thus, increase endogenous NO synthesis [95].

In addition to these discrepancies, the use of drugs that can modulate iNOS activity has caused viral immunopathogenesis and, thus, the occurrence of numerous side effects [24]. For this reason, new therapies have been explored and the main focus has been on gNO and NO-donor applications.

## 7. Therapeutic Potential of NO for the Treatment of COVID-19

### 7.1. Short Outline of SARS-CoV-2

Coronaviruses (CoVs) are a family of single-stranded positive-sense RNA viruses that contain a genome of approximately 30 kilobases and are named after their crown-like spike protrusions on the virus surface [96]. After crossing species barriers, CoVs trigger acute respiratory syndromes in humans, such as SARS or SARS-CoV-1 in 2002, MERS or MERS-CoV in 2012, and COVID-19 caused by SARS-CoV-2, in 2019 [97]. Although COVID-19 patients can be asymptomatic, those with symptoms suffer from fever, dry cough, shortness of breath and myalgia. Death is mainly caused by acute lung injury, ARDS and sepsis, which are caused by viral infection and are very similar to the pathological features of SARS and MERS [98]. According to genomic and proteomic analyses, the similarity between the total nucleotide sequences of SARS-CoV-2 and SARS-CoV-1 is about 79.5%, and the similarity of amino acid sequences in the seven replicate domains conserved in the open reading frame 1ab rises to 94.4% [99,100]. Consequently, SARS-CoV-2 belongs to the β-lineage of the coronavirus family and is a member of the SARS-CoV species [99,100]. It has been confirmed that SARS-CoV-2 and SARS-CoV-1 invade cells through a similar mechanism, i.e., by binding to the transmembrane type I receptor of human angiotensin-converting enzyme-2 (ACE-2) through protein S. The receptor-binding capacity of SARS-CoV-2 is approximately four times that of SARS-CoV-1 [101], which explains the higher infectivity of SARS-CoV-2.

Because of the overlap of genetic structures and pathological features between SARS-CoV-1 and 2, knowledge of SARS-CoV-1 has provided suggestions for understanding SARS-CoV-2, including the role, potential mechanism and therapeutic application of NO. The promising results from the rescue trial during the SARS-CoV-1-caused epidemic in the years 2002–2004 [50], prompted clinical trials during the SARS-CoV-2-caused pandemic in 2019 (COVID-19). To date, only Fang et al. [14] and Mir et al. [102] have attempted to systematically review the possible role of NO and its clinical use in the prevention and therapy of COVID-19.

### 7.2. Endogenous NO in COVID-19 Patients

There are four etiological pathways for COVID-19 that have been identified and described, and the level of endogenous NO and its bioavailability are quite low in each. *Pathway 1*—Although the main targets of SARS-CoV-2 are bronchial hair epithelial cells and type II lung cells, viral particles have also been found in endothelial cells. This is because SARS-CoV-2, SARS-CoV-1 and MERS-CoV lead to endothelial cell apoptosis with a consequent decrease in endothelial NO production [103]. *Pathway 2*—SARS-CoV-2 invades host cells via the binding of its surface glycoprotein-S to angiotensin-converting enzyme 2 (ACE2) [104]. The down-regulation of ACE2 has resulted in the inhibition of NO production and an increase in reactive oxygen species (ROS) production [105,106]. *Pathway 3*—NO/ROS imbalance in the body triggers the considerable release of proinflammatory cytokines and chemokines, which lead to the overstimulation of the inflammatory system, causing significant damage to tissues and organs. In the meantime, ROS production, especially in the mitochondria, generates mitochondrial dysfunctions because of increased mitochondrial membrane permeability [107]. *Pathway 4*—In order to restore the physiological ROS/NO balance, NO reacts with oxygen and its derivatives to form toxic reactive nitrogen species (RNS), such as peroxynitrite. The high concentration of ROS and RNS contributes to vascular oxidative stress, causing a change in vascular tones and leading to decreased NO bioavailability [108]. Therefore, most complications of COVID-19 disease are linked to inflammation and injury in the vascular system, in which NO plays a key role.

### 7.3. Applications of gNO-Based Therapy in COVID-19 Patients

On the basis of the outcomes obtained from the rescue clinical study following SARS-CoV-1 infection [14], gNO-based therapy was also investigated for treatment against SARS-CoV-2. Four clinical trials were initiated and are still ongoing.

In the first clinical trial (no. NCT04305457—17 February 2022, https://clinicaltrials.gov/ct2/show/NCT04305457), gNO is administered at 140–180 ppm concentration for 20–30 min, twice a day and for 14 consecutive days. The primary goal is to reduce the incidence of patients requiring intubation and mechanical ventilation, as a marker of deterioration from mild to severe COVID-19. The secondary goal is both the reduction of the mortality rate and the clinical recovery time. Clinical recovery means normalization of fever, respiratory rate, alleviation of cough and resolution of hypoxia. All these improvements must be maintained for 72 h. Other outcome measures are the increase in the percentage of patients with the negative conversion of RT-PCR from the oropharyngeal or oropharyngeal swab. The estimated study completion date is 1 April 2022.

The primary outcome of the second clinical trial (no. NCT04306393—17 February 2022, https://clinicaltrials.gov/ct2/show/NCT04306393) is to investigate whether gNO treatment can improve oxygenation in mechanically ventilated patients. The treatment involves inhalation of 80 ppm nitric oxide for 48 h, followed by 40 ppm, followed by weaning before stopping. If a patient dies during the first 48 h of treatment, the last available blood gas analysis will be used. Secondary outcomes are the calculation of the time to reach normoxemia, the percentage of SARS-nCoV-2 free patients during the first 28 days after enrollment, and the survival rate both 28 days and 90 days after treatment. The estimated study completion date is 21 December 2022.

The third clinical trial (no. NCT04312243—17 February 2022, https://covid-19.cochrane.org/studies/crs-13107176) focuses on hospital-associated transmission among healthcare workers. 470 healthcare workers scheduled to work with COVID-19 patients were randomly selected and divided into two groups: the first received nitric oxide gas (NO group, *n* = 235) while the second did not (control group, *n* = 235). The primary endpoint of this study is the incidence of subjects with COVID-19 disease 14 days after enrollment. Secondary endpoints are the percentage of caregivers presenting with a positive real-time RT-PCR test for SARS-CoV2 14 days after enrollment, the percentage of caregivers requiring quarantine, and the total number of days of quarantine in the two groups.

The fourth clinical trial (NCT03331445—17 February 2022, https://clinicaltrials.gov/ct2/show/NCT03331445), which ended 30 June 2021, aimed to investigate whether gNO therapy could reduce both mechanical ventilator intervention and oxygen therapy use [109,110,111,112,113]. This was an open-label safety study (COVID-19 Sub-study) with the primary outcome of measuring the safety of 160 ppm inhaled nitric oxide.

While other clinical trials have also been undertaken, none have released large-scale results as they were not representative of the majority of COVID-19 patients, or even of patients with a similar condition. Thus, gNO inhalation has not been promoted for the treatment of COVID-19. 

### 7.4. NO-Donors in COVID-19 Patients

To overcome the use of gNO therapy, Kalytera Therapeutics Inc. announced, on June 29, 2020, that it has entered into a binding Letter of Intent to license R-107 from the Salzman Group for the treatment of CoVs and COVID-19 infection. R-107 is a non-gaseous, liquid prodrug of NO. Following a single administration by injection, R-107 slowly releases NO into the lung tissues over 48 h (17 February 2022, https://stockhouse.com/news/newswire/2020/07/15/introducing-prodrug-that-s-anti-covid-19). Similarly, COViNOX is another NO-releasing drug that is in the third phase of clinical trials. In this trial, subjects were treated by means of an INOpulse device using an INOpulse nasal cannula (17 February 2022, https://www.clinicaltrials.gov/ct2/show/NCT04421508).

**Figure 4:**
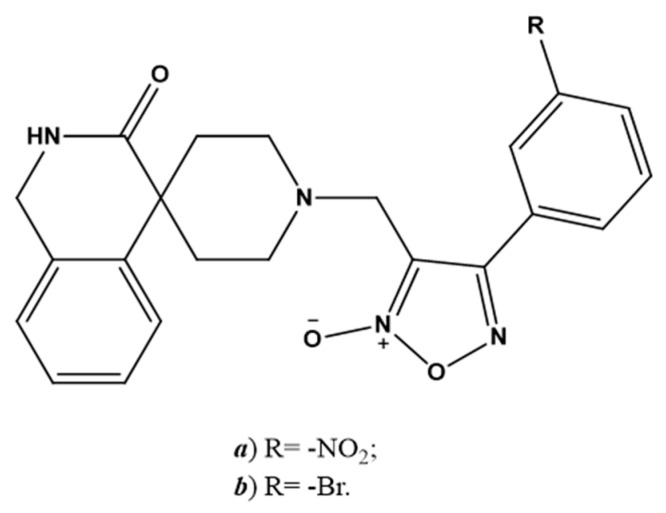
Chemical structure of NO-releasing inhibitors of SARS-CoV-2 proteases. (**a**,**b**) 4-phenyl furoxan derivatives.

SNAP (Figure 2e) has been investigated as a molecule belonging to the NO-donors class. Specifically, Akaberi et al. evaluated its antiviral effect in Vero E6 cells, which are an in vitro model of SARS-CoV-2 infection. SNAP was able to reduce the protease activity of SARS-CoV-2 by inhibiting its replication by up to 99.42% [23]. Two 4-phenyl furoxan derivatives (Figure 4a,b) are other promising NO-donors that can inhibit the main proteases of SARS-CoV-2 in silico [114].

## 8. Conclusions

Despite decades of studies, fighting viral infections is still a great medical challenge, as the SARS-CoV-2 spread has recently demonstrated. Since the discovery of NO, many studies have described its role on the respiratory system, even if some aspects still need clarification. NO improves oxygenation, regulates lung vasculature and surfactant function, improves ciliary motility and is involved in host defense.

As the role of NO in the defense against viral infections is becoming clearer and a positive role for this radical has been proposed in the treatment of respiratory infections, progress has been made in the development of NO-based therapies. gNO and NO-donors have been studied and some treatments have already been commercialized or are in ongoing clinical trials. Indeed, it should be considered that, depending on the concentration, NO can induce nitrosative stress and damage also host tissues.

In the case of gNO therapy, more studies are needed to define the amount of the radical that is able to reach the local tissues in order to maximize clinical outcomes while reducing the potential side effects. In the case of NO-donors, the use of nanosystems is a particularly promising field as it allows drug delivery and targeting to be improved. For this reason, further studies are important as they can contribute to the development of innovative therapeutic approaches.

## Figures and Tables

**Table 1:** Summaries of the studies on gNO and NO-donors as antiviral agents.

**Clinical Trials**			
**gNO Treatment**			
**Model**	**NO Dosage (ppm)**	**Duration**	**References**
SARS Coronavirus	<30 (stepwise)	3–7 days	[50]
**NO Donor Treatment**			
**Model**	**NO Dosage (ppm)**	**Duration**	**References**
Human Papillomavirus	Not specified	1–2 daily, 12 weeks	[51]
Molluscum Contagiosum	5% acidified nitrite donor in cream	1–83 months	[52]
	Not specified	1–2 daily, 12 weeks	[53]
**In-Vivo Studies**			
**gNO Treatment**			
**Model**	**NO Dosage (ppm)**	**Duration**	**References**
Human Influenza	80/160	Continuous/Intermittent	[54]
**NO Donor Treatment**			
**Model**	**NO Dosage (mM)**	**Duration**	**References**
Porcine Circovirus Type 2	10	1 daily, 6 days post-infection	[55]
**In-Vitro and Ex-Vivo Studies**			
**gNO Treatment**			
**Model**	**NO Dosage (ppm)**	**Duration (hrs)**	**References**
Human Influenza	80/160	3	[56]
Nosocomial pneumonia-related clinical isolates	200	2/6	[49]
**NO Donor Treatment**			
**Model**	**NO Dosage (μM)**	**Duration (hrs)**	**References**
Crimean Congo Hemorrhagic Fever Virus	50–400	24	[57]
Hantaan Hantavirus	100	12	[58]
Herpes Simplex Virus and Vaccinia Virus	1000	12	[59]
Human Influenza	50–400	24	[60]
Human Papillomavirus	Not specified	1	[61]
Human Rhinovirus	100–1000	4/24	[62,63,64]
SARS Coronavirus-1	65–500	72	[16]
SARS Coronavirus-2	20–500	36–72	[23]
